# Evaluation of subcutaneous and alveolar implantation surgical sites in the study of the biological properties of root-end filling endodontic materials

**DOI:** 10.1590/S1678-77572010000100013

**Published:** 2010

**Authors:** Luciano Tavares Angelo CINTRA, Pedro Felício Estrada BERNABÉ, Ivaldo Gomes de MORAES, João Eduardo GOMES-FILHO, Tetuo OKAMOTO, Alberto CONSOLARO, Tiago Novaes PINHEIRO

**Affiliations:** 1 DDS, MSc, PhD, Assistant Professor, Department of Restorative Dentistry, Maringá Dental School, UNINGÁ University, Maringá, PR, Brazil.; 2 DDS, MSc, PhD, Full Professor, Department of Operative Dentistry, Araçatuba Dental School, São Paulo State University, Araçatuba, SP,Brazil.; 3 DDS, MSc, PhD, Associate Professor, Department of Operative Dentistry, Endodontics and Dental Materials, Bauru School of Dentistry, University of São Paulo, Bauru, SP, Brazil.; 4 DDS, MSc, PhD, Associate Professor, Department of Operative Dentistry, Araçatuba Dental School, São Paulo State University, Araçatuba, SP,Brazil.; 5 DDS, MSc, PhD, Full Professor, Department of Stomatology, Bauru School of Dentistry, University of São Paulo, Bauru, SP, Brazil.

**Keywords:** Root-end filling endodontic materials, Mineral Trioxide Aggregate, Animal model, Biocompatibility

## Abstract

**Objective:**

The aim of this study was to compare two methodologies used in the evaluation of tissue response to root-end filling materials in rats.

**Material and Methods:**

Forty rats were divided into 4 groups: in Groups I and II (control groups), empty polyethylene tubes were implanted in the extraction site and in the subcutaneous tissue, respectively; in Groups III and IV, polyethylene tubes filled with ProRoot MTA were implanted in the extraction site and in the subcutaneous tissue, respectively. The animals were killed 7 and 30 days after tube implantation, and the hemi-maxillas and the capsular subcutaneous tissue, both with the tubes, were removed. Specimens were processed and evaluated histomorphologicaly under light microscopy. The scores obtained were analyzed statistically by the Kruskal-Wallis test (p<0.05).

**Results:**

There were no statistically significant differences between the implantation methods (p=0.78033, p=0.72039). It was observed that the 30-day groups presented a more mature healing process due to smaller number of inflammatory cells.

**Conclusion:**

The present study showed no differences in tissue responses as far as the implantation site and the studied period were concerned. Alveolar socket implantation methodology represents an interesting method in the study of the biological properties of root-end filling endodontic materials due to the opportunity to evaluate bone tissue response.

## INTRODUCTION

Several methods, such as animal teeth^12,25,33^, subcutaneous implantation^12,21,23,35,36^, alveolar sockets^7,8^, and culture cells^18,26^, have been used in the evaluation of biocompatibility.

Root-end filling material have been evaluated by use of numerous in vivo methodologies^7,8,11,18,21,33,37^, including rat subcutaneous tissue implantation, which is recommended by the Council on Dental Material and Devices, according to ISO 7405 standard^2,14^. Polyethylene or dentin tubes are filled with test materials and implanted in the subcutaneous tissue in the back of rats, with the purpose of studying the inflammatory tissue response evoked^35,36^. Stanford^27,28^ reported standards for biological tests of dental materials, including subcutaneous implantation. Olsson, et al.^21^ and Stanley^29^ considered subcutaneous implantation as a secondary test in the biological evaluation of dental materials.

Subcutaneous implantation has been used as an established method since the results obtained by Torneck, et al.^35,36^. However, another methodology was introduced by Degrood, et al.^8^ in which implantations of polyethylene tubes filled with amalgam or Ketac Fill were performed in mandibular alveolar sockets. Similar methodology was used by Cintra, et al.^7^ with the difference that the site employed to compare MTA and MBPc were maxillary alveolar sockets. Nary Filho and Okamoto^20^ also considered these sites as adequate for evaluating the tissue response to tubes filled with biomaterials.

Alveolar socket implant presents specific features regarding the different stages of healing maturation^4,15,17^. Another important aspect is that the alveolar socket represents a bone cavity covered by periodontal ligament, a special connective tissue, extremely important for clot organization in dental extraction wounds^4,17,20^. Therefore, this study model offers an interesting environment to simulate what occurs in endodontic therapy and apical surgery sites^7^. The methodology proposed by Degrood, et al.^8^ seems to be an adequate alternative to subcutaneous implant, although there is a lack of comparative studies to sustain this hypothesis.

Regarding the root-end filling endodontic materials, many studies have been published to determine the best root-end filling material regarding the physical and biological properties^1,3,7,8,11,12,17,24,25,30,32,33^. Upon review, it was observed that among the materials, amalgam, gutta-percha, composites, glass ionomers and zinc oxide cements (IRM and Super EBA) are the most frequently used.

Recent studies demonstrated that MTA has an interesting behavior in the apical tissues. It has demonstrated adequate sealing ability against microorganisms and its products^1,30^, biocompatibility and adequate solubility property in the mouth fluid^9,^ as well as dimensional stability and radiopacity^32^.

The evaluation of methodologies to study the biological properties of root-end filling endodontic materials applied in the present study include empty polyethylene tubes established by Tornek^35,36^ and polyethylene tubes filled with MTA. The last was chosen as test material due to its biological properties studied recently in the literature^1,3,5,7,9-13,16,19,30-34,37^.

The aim of this study was to compare the alveolar sockets and the subcutaneous implantation methodologies in order to evaluate the biological properties of root-end filling endodontic materials.

## MATERIAL AND METHODS

Forty Wistar rats were intramuscularly anesthetized with ketamine hydrochloridre (87 mg/kg) and xylazine (13 mg/kg) and divided into four groups, with 10 animals for two period of time: Group I, empty polyethylene tubes (Embramed Ind. Comércio Ltda., São Paulo, SP, Brazil; 1.0 mm internal diameter x 1.67 mm external diameter x 3.0 mm length) were implanted in the alveolar sockets; Group II, empty polyethylene tubes were implanted in the subcutaneous tissue; Group III, polyethylene tubes filled with Pro Root^TM^ MTA (Dentsply, Tulsa, OK, USA) were implanted in the alveolar sockets; Group IV, polyethylene tubes filled with Pro Root^TM^ MTA were implanted in the subcutaneous tissue.

**Figure 1:**
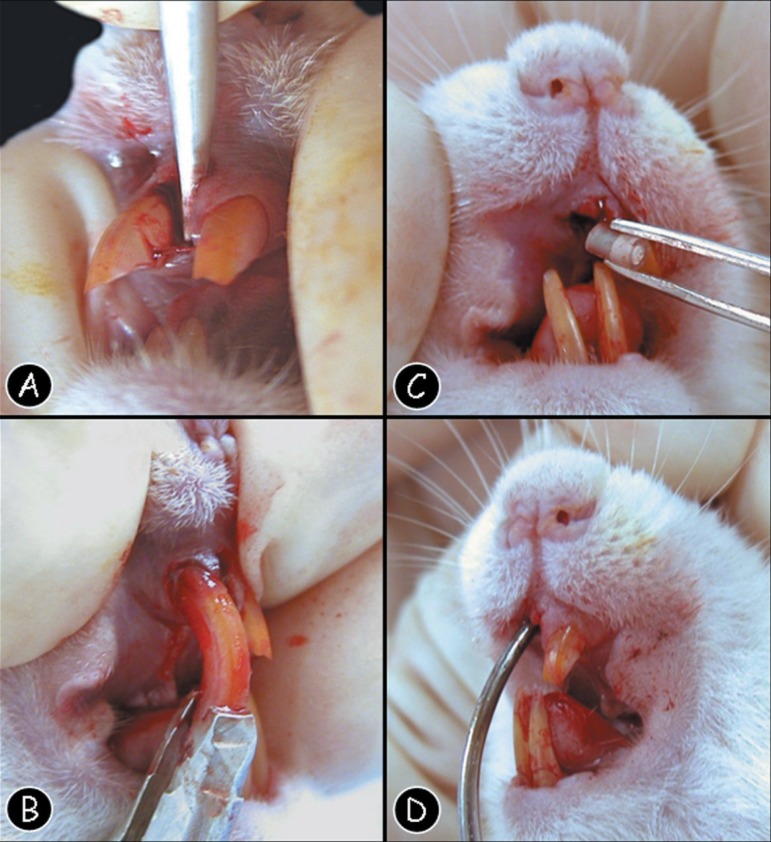
(A) Surgical aspect of the alveolar luxation performed with a special instrument inserted between the tooth and the alveolar cortical bone. (B) Surgical aspect of the tooth extraction. Note that a special instrument is placed in the dental socket for the right superior incisive extraction. (C) Implantation of the polyethylene tubes, filled with the tested material, inside the alveolar socket using tweezers. (D) Introduction of the polyethylene tubes in the alveolar middle third with a specially adapted instrument

**Figure 2:**
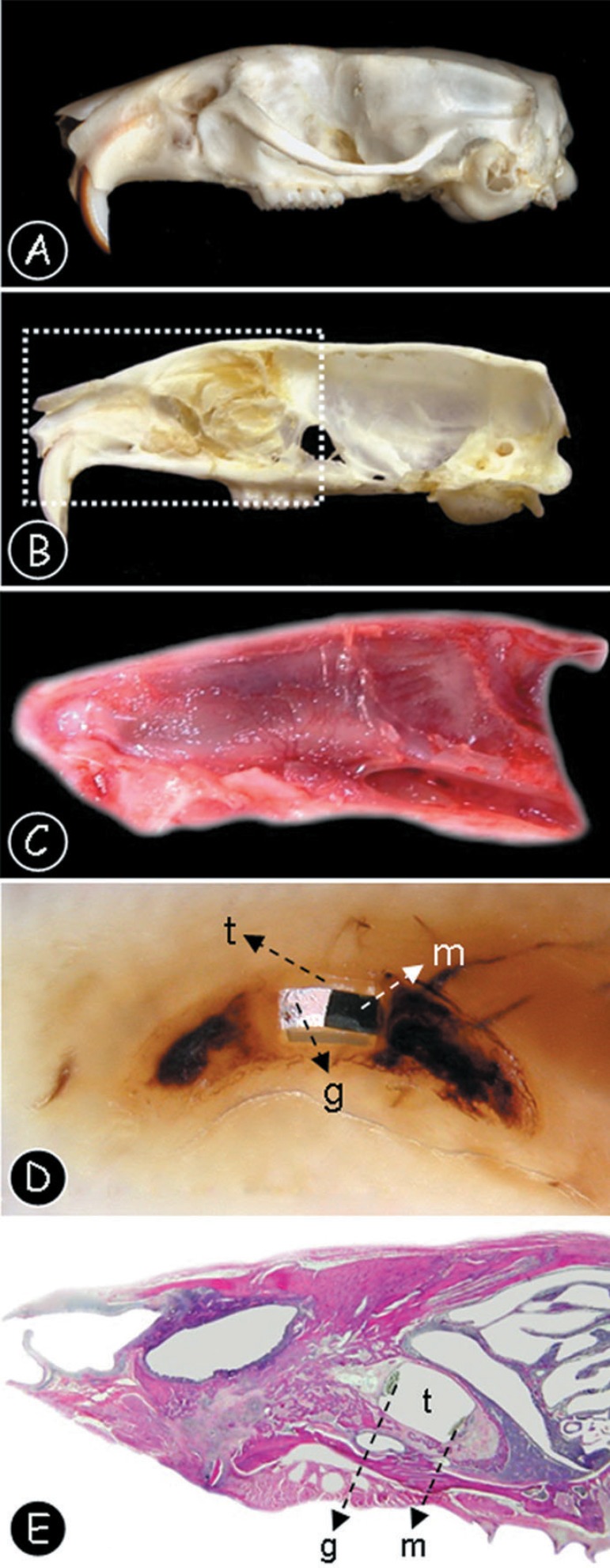
(A) Lateral view of the rat skull anatomy after maceration procedures. (B) Medium sagital section of the rat skull, after the nasal septum excision (C) Lateral view of the right hemi-maxilla after surgical removal. (D) Longitudinal section of the dental alveolus after paraffin inclusion. Note that the polyethylene tube is present (t), filled with MTA (m) and the sealing material (g). (E) Panoramic tissue section of the right hemi-maxilla after hematoxylin and eosin staining. Note that the polyethylene tube is present (t), filled with MTA (m) and the sealing material (g)

Eighty polyethylene tubes were used for implantation in the subcutaneous tissue and the alveolar sockets of the rats. Each tube had one end sealed with a lightly heated 1.0 mm-thick layer of gutta-percha (Odahcam; Herpo Produtos Dentários Ltda, Rio de Janeiro, RJ, Brasil). The gutta-percha was condensed into the tube with a cold gutta-percha condenser to avoid the deformation of the polyethylene tubes walls. The remaining 2.0 mm were filled with the Pro Root^TM^ MTA. This procedure prevents fresh MTA to extrude the opposite extremity. The control tubes remained totally empty. Each animal had its right upper incisor extracted using special instruments^7,20^ for subluxation and extraction. After tooth extraction and hemostasis, the tubes were placed in the apical third of the alveolar socket with a carrier, so that the MTA would always face towards its base according to the study of Cintra, et al.^7^ (Figures 1 and 2). Fresh MTA was handled according to manufacturer’s instruction, and used to fill sterile tubes. Each animal received two polyethylene tubes: one tube in the alveolar socket and another in the subcutaneous tissue of the dorsum. The gingival tissue and tissue of the dorsum were sutured over the extraction socket with non-resorbable silk 4-0 sutures (Ethicon, Johnson & Johnson Ind. Comércio Ltda., São José dos Campos, SP, Brasil).

The animals were killed 7 and 30 days after implantation of the tubes, with heart anesthetic injection. Hemi-maxilla with the alveolar sockets and tubes were removed, fixed in 10% formalin and decalcified in 10% EDTA. The specimens from subcutaneous tissue were fixed only in 10% formalin. All specimens were washed in running water, dehydrated in an increasing series of etahnol concentrations, cleared in xylol, and embedded in paraffin. Six-micrometer-thick sections were obtained and stained with hematoxylin and eosin for analysis under light microscopy.

Inflammatory cells were evaluated in relation to extension and intensity by calibrated evaluators. The scores used were in accordance with Örstavik and Mijör^22^ and Cintra, et al.^7^ (Figure 3) and were subjected to statistical analysis by the Kruskal-Wallis test (α=0.05).

**Figure 3:** Criteria for scoring inflammatory tissue response

	**Scores**	**Extent**	**Verbal Descriptions**
Inflammatory response	1	Absent	Thickness of reaction zone similar or only slightly wider than along side tube; none or few inflammatory cells.
	2	Mild	Increased reaction zone; presence of macrophages and/or plasma cells.
	3	Moderate	Increased reaction zone; presence of macrophages and plasma cells; occasional foci of neutrophil granulocytes and/or lymphocytes.
	4	Severe	Focal areas of necrosis; tissue densely infiltrated by inflammatory cells.

## RESULTS

Comparative analysis of the morphologic features observed in the studied groups showed that, the 7-day groups for both methodologies presented moderate or discrete inflammatory response. On the other hand at the 30-day studied groups, it was possible to grade most of the inflammatory reactions as discrete or absent (Figure 4).

**Figure 4:** Inflammatory scores related to the groups

		**7 Days**				**30 Days**			
		**I**	**II**	**III**	**IV**	**I**	**II**	**III**	**IV**
Inflammatory response	1- Absent	0/10	0/10	0/10	0/10	3/10	1/10	2/10	2/10
	2- Mild	7/10	7/10	6/10	5/10	6/10	7/10	7/10	6/10
	3- Moderate	3/10	3/10	4/10	5/10	1/10	2/10	1/10	2/10
	4- Severe	0/10	0/10	0/10	0/10	0/10	0/10	0/10	0/10

**Figure 5:**
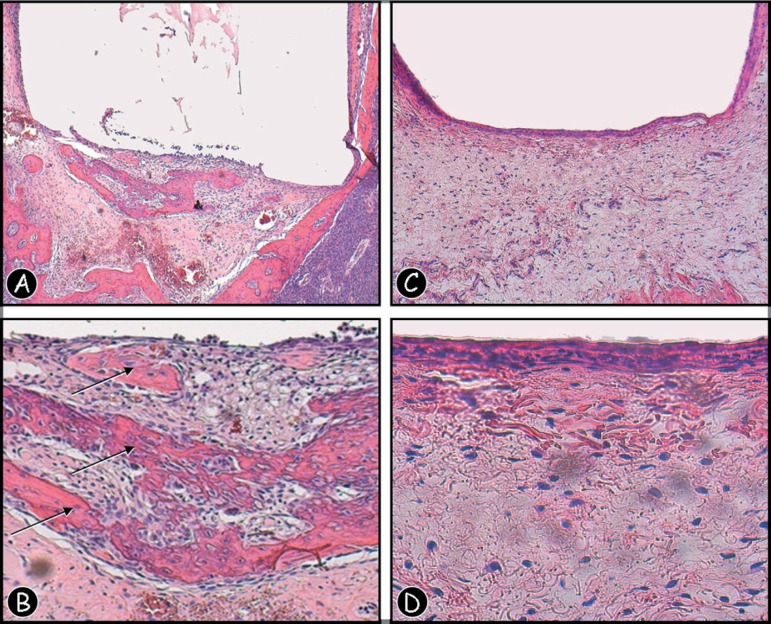
(A,B) Photomicrograph of a section of rat dental socket from 30-day control group (empty polyethylene tube) specimen, showing mild inflammatory infiltrate and new bone formation (arrows) with newly formed blood vessels of several diameters [Hematoxilin and eosin (H&E), original magnification ×50 and X 250]. (C,D) Photomicrograph of a section of rat subcutaneous tissue from 30-day control group (empty polyethylene tube) specimen, showing mild inflammatory infiltrate with neutrophils, lymphocytes, and giant cells (H&E, original magnification ×50 and ×250)

The 7-day control group revealed newly formed organized granulation tissue with discrete inflammatory response, young fibroblasts, few macrophages and lymphocytes, observed in both of the studied sites. The 30-day control group, presented more organized connective tissue in the sockets, and a neoformed revasculated bone tissue (Figure 5A and 5B). Regarding the subcutaneous implantation site, the 30-day group reveled absence of mineralized tissue Figure 5C and 5D).

The features of inflammatory infiltrate were similar between groups with tubes filled with MTA and the empty tube groups for all periods of observation.

**Figure 6:**
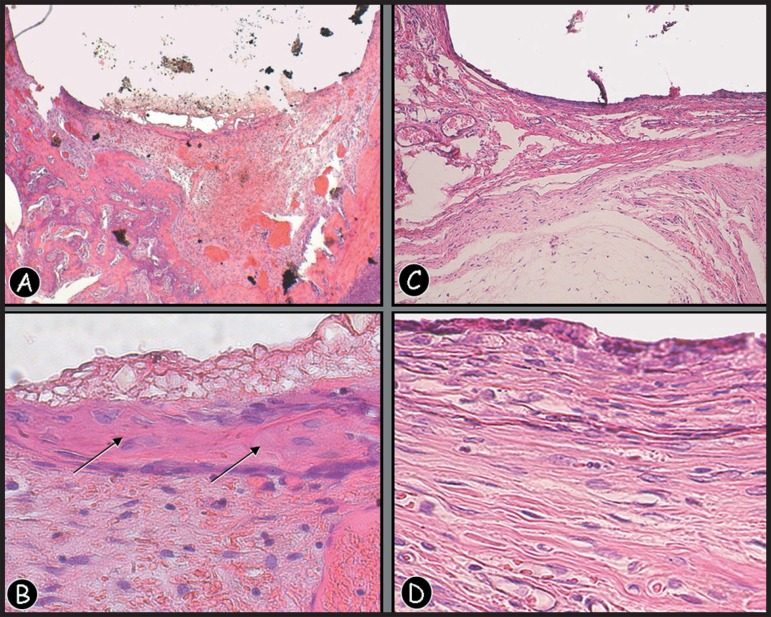
(A,B) Photomicrograph of a section of rat dental socket from 30-day MTA implant specimen, showing mild inflammatory infiltrate, new bone formation and dystrophic calcifications close to the material (arrows) [Hematoxilin and eosin (H&E), original magnification ×50 and X 250]. (C,D) Photomicrograph of a section of rat subcutaneous tissue from 30-day MTA implant specimen; well organized granulation tissue showing a mild inflammatory infiltrate, young fibroblasts, few macrophages and lymphocytes (H&E, original magnification ×50 and ×250)

The 7-day MTA group presented a superficial layer with irregular thickness, highlighting coagulation necrosis on both sites of implantation. The 30-day evaluation of alveolar socket group showed irregular basophilic areas present subjacent to the material, suggesting that, with the increase of the studied period, the site could become a matrix for mineralization (Figure 6A and 6B). The subcutaneous tissue group revealed well organized granulation tissue, showing a mild inflammatory infiltrate, young fibroblasts, few macrophages and lymphocytes as well (Figure 6C and 6D).

The groups did not show statistically significant differences for any period of time (p=0.78033, p=0.72039). However, it was observed that all 30-day groups presented a more mature healing process due to the smaller number of inflammatory cells.

## DISCUSSION

The usual way to test biological material in alveolar sockets wounds consists of the implantation of the tested material directly in the surgery site. However, when it comes to the study of root-end filling materials biological characteristics, many variables need to be considered, such as site of implantation, amount of material, powder/liquid proportion, contact mechanism between the material and the biological system, environment temperature, and working time, which are difficult to be standardized.

In an attempt to control the presence of these variables, this work incorporated one component of the rat subcutaneous implantation methodology, the polyethylene tubes, as performed in previous studies^7,8^. The use of polyethylene tubes filled with endodontic materials in biocompatibility tests is widely accepted^2,27,29^.

With the use of polyethylene tubes, it is possible to standardize the amount of material to be implanted in each specimen, and to limit the contact area between the material and the tissue. Therefore, the methodologies of comparative analysis could be used in similar conditions.

The results showed normal healing in close contact with the tube walls, in both methodologies. These evidences indicate that polyethylene tubes are biocompatible with alveolar sockets and subcutaneous tissues, already confirmed by other reports^7,8,21,23,35,36^. It was also observed a connective tissue growth inside the polyethylene tubes in both methodologies, as observed by Torneck, et al.^35,36^.

According to Astrand and Carlsson^4^ and Lamano Carvalho, et al.^15^, the complete healing process in alveolar socket wounds takes up to 27-30 days. The present study showed incomplete healing at the 30-day period with empty or filled tubes. This aspect suggests that the physical presence of the polyethylene tubes *per se* delays the healing process. These findings justify the use of a control group with empty tubes.

With regard to the tissue response to Pro Root^®^ MTA, it was observed that its irritating potential, in both methodologies, was similar to the findings of Holland, et al.^12^, in subcutaneous tissue, highlighting an organized connective tissue with mineralized tissue and few inflammatory cells. Fibrous capsule and inflammatory cells were observed by Yaltirik, et al.^37^, with implantation of polyethylene tubes filled with MTA in rat subcutaneous tissue. The authors also noted dystrophic calcifications close to the material when the specimens though Von Kossa staining technique. Similar morphologic findings were found in the present study regarding the mineralized tissue formation using HE stain, as shown in Figure 6A and 6B).

MTA was able to promote a favorable environment for the formation of mineralized tissue in close contact with the material, which was not observed with the empty tubes. These observations were reported in other studies using HE or Von Kossa stain methods^12,13^.

The largest period of observation was 30 days, different of that used by DeGrood, et al.^8^. Maybe in a longer time-period, the healing process in all specimens would present no inflammatory cells, as it was seen in some of the specimens of the present study on the 30th day.

The histological processing of the alveolar socket specimens was performed very much alike the subcutaneous tissue specimens, except for the removal of the polyethylene tubes. The alveolar socket implantation methodology allows microtome slicing without the tube removal, probably due to the anchorage provided by the tube walls on the bone tissue, even after the demineralization process. The subcutaneous implantation allows the migration of the tubes, which could be seen during the removing. On the other hand, the implantation in alveolar sockets maintains the tube in position, thus offering more reliable results.

The odontogenic environment is another important point to be taken into consideration, as far as results are concerned. In the alveolar sockets, the materials were kept in contact with tissues, representing the more natural condition in which the endodontic materials are normally employed in human beings, therefore the results are closer to those expected in vivo.

One of the major shortcomings of this study is the fact that in vivo studies that have investigated tissue responses or biocompatibility to root-end filling materials are unrepresentative of the typical clinical situation if surgery is performed under ideal circumstances and in infection-free roots. In order to simulate better the true clinical situation of an infected root canal and to study the tissue responses to potential root-end filling materials, an experimental animal model of infected teeth with periradicular inflammation must be considered^6^. Another major drawback of this study is the fact that both control and experimental groups demonstrated similar tissue responses. Even the control group, where the tubes where implanted in the extraction sites showed bone formation.

The quantitative analysis, performed by means of grading scores for the magnitude of the microscopic phenomena observed, was based on the criteria used by ?rstavik and Mijör^22^ and Cintra, et al.^7^.

## CONCLUSION

The present study showed that there were no differences between the tissue responses as far as implantation site and studied period are concerned. Alveolar socket implantation methodology represents an interesting method in the study of the biological properties of rootend filling endodontic materials due to the opportunity to evaluate the bone tissue response.
